# Experimental and clinical studies on radiation and curcumin in human glioma

**DOI:** 10.1007/s00432-020-03432-2

**Published:** 2020-10-28

**Authors:** Peter Sminia, Jaap van den Berg, Arthur van Kootwijk, Eline Hageman, Ben J. Slotman, Wilko F. A. R. Verbakel

**Affiliations:** Department of Radiation Oncology, Amsterdam University Medical Centers, VU University, Cancer Center Amsterdam, De Boelelaan 1117, 1081 HV Amsterdam, The Netherlands

**Keywords:** Curcuma longa, Curcumin, Radiation, GBM, Nanoparticles, Radiosensitization

## Abstract

**Purpose:**

There is progressing evidence for the anti-cancer potential of the natural compound and dietary spice curcumin. Curcumin has been ascribed to be cytotoxic for various tumour cell types, to inhibit cell proliferation and to interfere with the cellular oxidant status. The compound has been notified as a therapeutic agent with radiosensitizing potential in brain tumour therapy. We considered the rationale to combine curcumin with radiation in the treatment of human glioblastoma multiforme (GBM).

**Method:**

Determination of clonogenic cell survival following exposure of U251 human glioma cells to single dose (1–6 Gy) and fractionated irradiation (5 daily fractions of 2 Gy) without and with curcumin. Additional literature search focused on the interaction between curcumin and radiotherapy in experimental and clinical studies on human glioma.

**Results:**

No interaction was found on the survival of U251 human glioma cells after irradiation in combination with curcumin at *clinically achievable concentrations*. Experimental in vitro and in vivo data together with clinical bioavailability data from the literature do not give evidence for a radiosensitizing effect of curcumin. Reported GBM intratumoural curcumin concentrations are too low to either exert an own cytotoxic effect or to synergistically interact with radiation. Novel approaches are being explored to increase the bioavailability of curcumin and to facilitate transport over the blood–brain barrier, aimed to reach therapeutic curcumin levels at the tumour site.

**Conclusion:**

There is neither a biological nor clinical rationale for using curcumin as radiosensitizer in the therapy of GBM patients.

## Introduction

Glioblastoma multiforme (GBM) is the most malignant and common human brain tumour. GBM patients are generally treated according to the current standard protocol of surgery followed by radiotherapy and concomitant and adjuvant chemotherapy, typically with the alkylating agent temozolomide. Despite this aggressive multimodality therapy, patients’ survival at 5 years after diagnosis is still only a few percent (Stupp et al. 2009). The poor treatment response of GBMs has been ascribed to radioresistant glioma stem cells and phenotypic heterogeneity.

A large number of studies have revealed that curcumin, the principle ingredient of the Indian dietary spice turmeric (*Curcuma longa*), demonstrates anti-cancer properties in a variety of tumour types, including GBM (Hatcher et al. 2008). Curcumin, which is also known for its anti-inflammatory and oxidant activity, affects a wide range of cell-signaling pathways, resulting in inhibition of cell proliferation and induction of apoptosis. Despite this, the therapeutic application of curcumin is delimited due to its poor intestinal absorption and pharmacokinetics (Schiborr et al. 2014; Nelson et al. 2017). Following repeated intake of curcumin in humans, blood serum concentration peaked at approximately 2 μM (Cheng et al. 2001), which might be too low for anti-cancer efficacy. Mean intratumoural curcumin concentrations of approximately 0.15 μM have been reported after orally administered micellar curcuminoids to GBM patients (Dutzmann et al. 2016).

Since radiotherapy belongs to the standard treatment of GBM patients, the present study is focused on the radiosensitizing potential of curcumin in human glioma. Experimental data on the radiosensitizing potential of curcumin in U251 human glioma cells are presented. Furthermore, a literature overview is given about preclinical and clinical data on the use of curcumin additional to radiotherapy in the treatment of gliomas. Of particular importance is the dose range and timing of curcumin administration when combined with radiation and the mechanism of interaction between both modalities. Novel technical approaches to increase the bioavailability of curcumin and/or to facilitate its transport over the blood–brain barrier, aiming to reach therapeutic levels in GBMs, are discussed. Finally, in view of available preclinical and clinical data, the use of curcumin in brain tumour therapy is debated.

## Materials and methods

### Cell culture

U251 human glioma cells, obtained from ATCC, were cultured in Dulbecco’s Modified Eagle’s Medium (DMEM) supplied with 10% fetal calf serum (FCS), 1% penicillin, and streptomycin (Invitrogen, Groningen, The Netherlands), and incubated at 37 °C in a 5% CO2 humidified atmosphere. Cells were routinely passaged and free from mycoplasm.

### Drug and radiation treatments

**Fig. 1:**
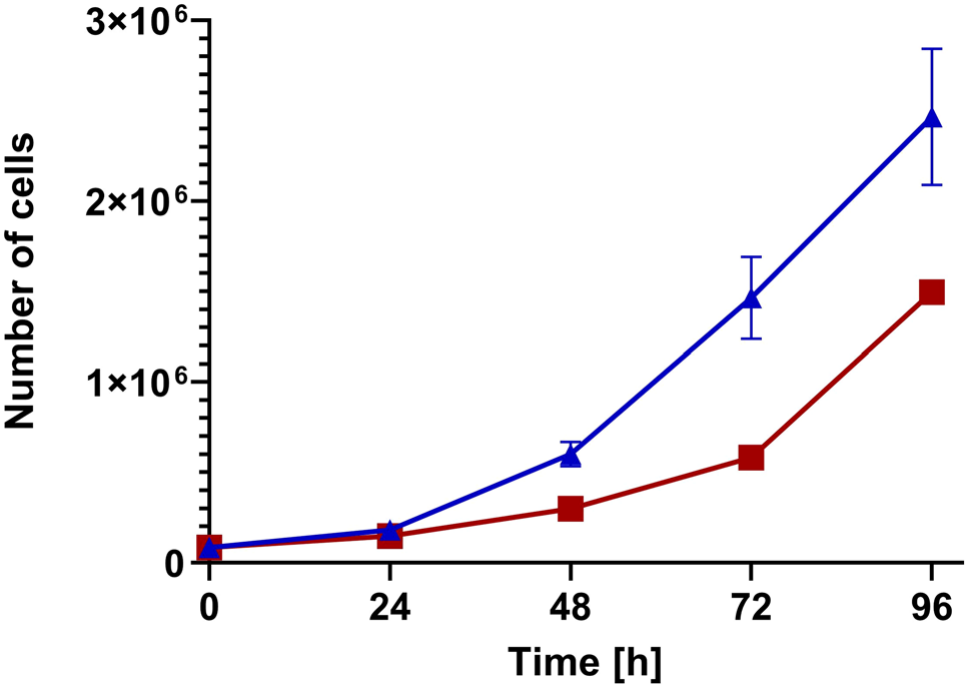
Cell proliferation ± curcumin. Proliferation of U251 glioma cells after up to 96 h continuous exposure to 5 μM curcumin (square) relative to control (triangle)

Curcuma Longa (MW 368.38; Sigma Aldrich, St. Louis, MO) was diluted in DMSO, to a 20 mM stock solution, and added to the cells at indicated final concentrations. 0.1% DMSO solvent was used as control. Cells were γ- irradiated using a Gammacell 220 (MDS Nordion, Canada) at a dose rate of approximately 180 Gy/h Fig. 1.

### Clonogenic assay

**Fig. 2:**
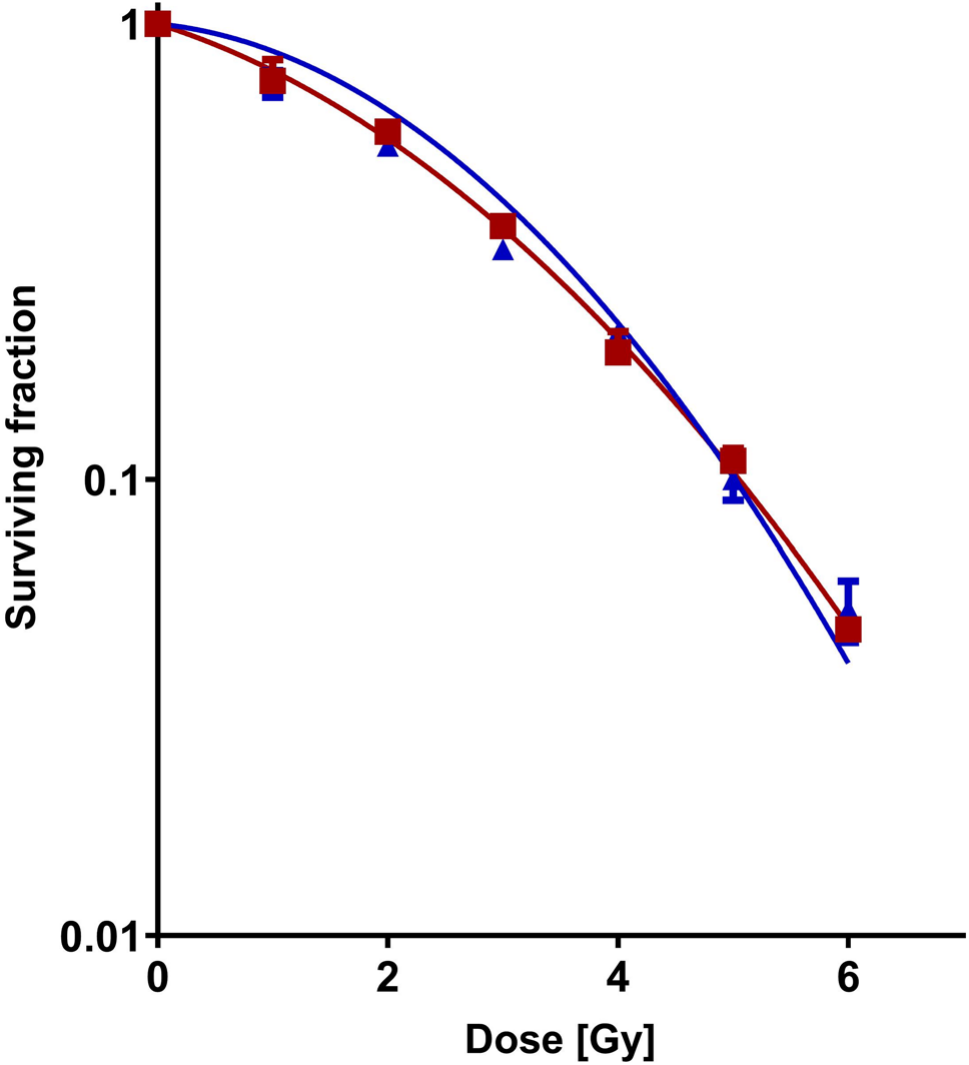
Single-dose irradiation ± curcumin. Clonogenic survival of U251 glioma cells after exposure to 5 μM curcumin for 72 h combined with single-dose irradiation (square) relative to control (triangle)

Survival of U251 cells following treatment was evaluated by the clonogenic assay [37]. Dose finding experiments were performed using curcumin concentrations up to 100 μM and exposure times between 0.5 and 96 h. On basis of the data (see Results section), 5 μM curcumin was used in the combination experiments with either single dose (0–6 Gy) or fractionated (5 daily fractions of 2 Gy) irradiation. Curcumin was administered prior or concomitant with irradiation for different durations (24–96 h). Following treatment, cells were grown for 10 days, fixed with 70% ethanol for 30 min, and stained with Giemsa solution (Merck, Darmstadt, Germany). Colonies containing > 50 cells were counted visually and plating efficiency (PE) was calculated by dividing the number of colonies counted by the number of colonies seeded. Cells were fixed and colonies were counted. Surviving fractions (SF) were calculated by dividing the PE of treated cells by the PE of controls. Experiments were performed in duplicate for each cell line. Cell survival curves presented in Fig. 2 were generated using the LQ model (Franken et al. 2006) and normalized for the effect of curcumin alone. Figures were drawn using GraphPad Prism version 8.0.2 for Windows (GraphPad Software, San Diego, California USA).

### Literature search

A literature search was performed on PubMed (https://www.ncbi.nlm.nih.gov/pubmed) using the search terms: curcumin, radiation, and brain tumour.

## Results

**Fig. 3:**
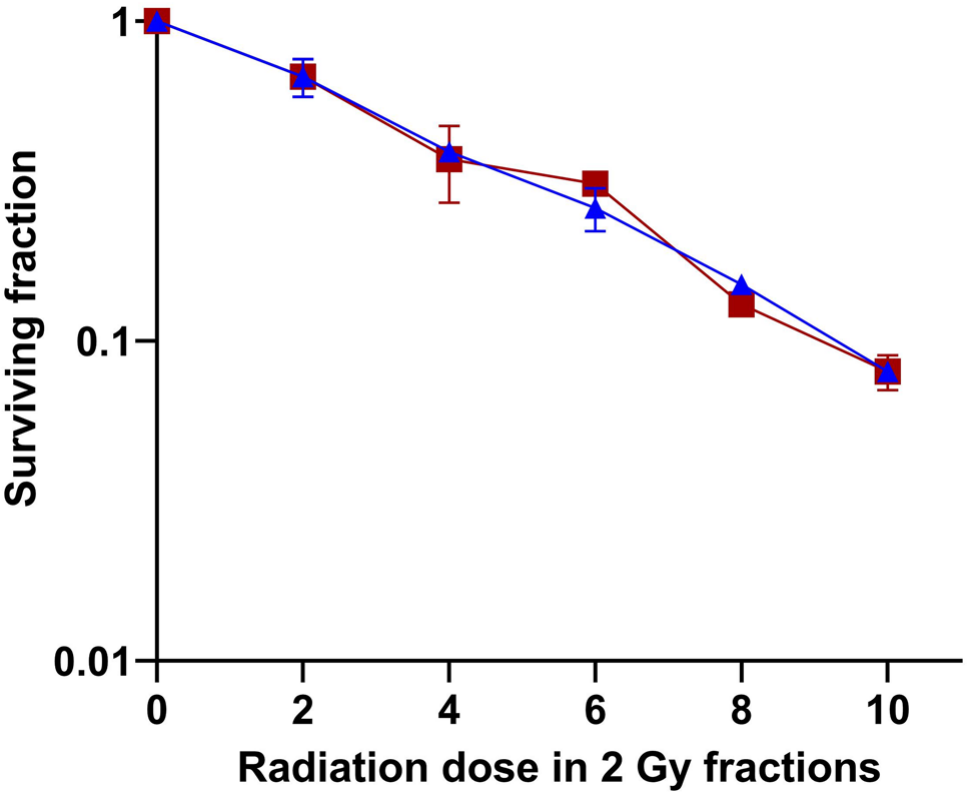
Fractionated irradiation ± curcumin. Clonogenic survival of U251 glioma cells with (square) and without (triangle) 5 μM curcumin for 72 h during fractionated irradiation with five daily fractions of 2 Gy

The optimal dose and exposure duration for investigation of the radiosensitizing effect of curcumin were derived from the curcumin dose–response effect evaluation. Dose escalation and exposure duration experiments were performed using curcumin concentrations up to 100 μM with exposure times ranging from 0.5 to 96 h. Three pilot experiments investigating the effect on cell proliferation and clonogenic cell survival of (1) long-term (96 h) exposure and (2) short-exposure duration (2 h) to a range of curcumin doses and (3) 0–3 h to 100 μM curcumin. Figure 1 demonstrates that 96 h exposure to a 5 μM curcumin inhibited the proliferation of U251 cells, while cell survival was non-significantly affected. Curcumin at doses > 5 μM for 96 h and doses beyond 25 μM for 2 h as well as 100 μM curcumin for > 0.5 h were found to reduce the survival of U251 cells (data not shown). To study the interaction between curcumin and irradiation on the survival of U251 cells, a curcumin exposure at a dose of 5 μM for 72 h was combined with single-dose irradiation. Clonogenic cell survival curves of irradiation alone and in combination with curcumin clearly show the absence of interaction between both treatment modalities (Fig. 2). Cell survival data presented in Fig. 3 confirm the absence of a radiosensitizing effect: no interaction was found on U251 cells exposed to 5 μM curcumin for 72 h concomitant with five daily fractions of 2 Gy.

## Discussion

### Preclinical GBM studies of curcumin alone and in combination with irradiation

Klinger and Mittal (2016) reviewed the biological effects of curcumin, with emphasis on its prospective benefit for brain tumour therapy. The anti-cancer effects of curcumin include modulation of cell proliferation, induction of apoptosis, inhibition of angiogenesis, autophagy induction, stimulation of the immune response, as well as inhibition of cell invasion and metastasis. Curcumin, in the dose range of 10–20 μM, suppressed E3 ubiquitin–protein ligase NEDD4 (Neural precursor cell Expressed Developmentally Downregulated protein 4) which is overexpressed in gliomas, leading to inhibition of cell proliferation, apoptosis, cell migration, and invasion (Wang et al. 2017). Zanotto-Filho et al. (2012) studied the effects of curcumin on the growth of glioma cells in vitro and in a preclinical glioma model in vivo. Curcumin inhibited cell proliferation and cell migration and induced cell death. They found that curcumin decreased cell viability in four malignant glioma cell lines at IC50 between 19 and 28 μM. Healthy astrocytes were much less sensitive to curcumin, with an IC50 of 135 μM after exposure during 36 h. Therewith, they confirmed experimental data by Dhapani et al. (2007) who also demonstrated that primary cortical neurons and astrocytes can tolerate high doses of curcumin, up to 50 μM for 48 h. In vivo, curcumin administration significantly reduced the size of intracranially growing GBMs in rats, while no evidence of healthy tissue toxicity was observed (Zanotto-Filho et al. 2012).

Rodriguez et al. (2016) analysed a total of 19 in vitro and 5 in vivo studies on the therapeutic role and molecular biology of curcumin in the treatment of GBM. All studies indicate a decrease in cell viability through various pathways. Curcumin inhibits the expression of pro-survival proteins, such as NFkB, AP-1, and PI3K, and activates apoptotic pathways via p21, p53, caspase 3. Furthermore, it has been demonstrated that sub-toxic levels of curcumin (2.5 μM) induced Reactive Oxygen Species (ROS) which could in particular damage glioma stem cells, therewith inhibiting cell proliferation, sphere formation, and colony formation (Gersey et al. 2017). This latter assay was performed with the continuous presence of curcumin, and hence, observed effects are all due to inhibition of cell proliferation. Induction of ROS is possibly mediated through downregulation of the STAT3 protein, which might enhance the effect of radiation. Inhibition of ROS with the anti-oxidant N-acetylcysteine reversed the effects, indicating an ROS-dependent mechanism (Gersey et al. 2017).

Taken together, curcumin alone seems to have a broad anti-cancer effectiveness via different molecular pathways, finally resulting in inhibition of cell migration and cell proliferation, and even in apoptotic cell death. A drawback of most studies is that effects were observed at relative high doses of curcumin in an IC50 range between 12 and 97 μM. However, at lower curcumin concentrations, the compound might interact with other anti-cancer modalities, like chemotherapeutic agents and radiation (Li et al. 2007; Hussaarts et al. 2019). For that reason, and because radiotherapy is the main part of treatment of GBM patients, the present study focused on the radiosensitizing potential of curcumin.

Preclinical GBM studies on the combination of curcumin with irradiation are depicted in Table 1. Dhandapani et al. (2007) treated T98G and U87MG glioma cells with curcumin alone for 6 h (25 μM), irradiation alone (single dose of 5 Gy), and their combination. Their data show a synergistic effect of curcumin and irradiation, which was ascribed to inhibition of anti-apoptotic gene expression, resulting in mitochondrial dysfunction and oxidative cell damage and therewith in cell death (Dhandapani et al. 2007).

**Table 1:** Studies on the combination of curcumin with irradiation in various in vitro and in vivo GBM models

In vitro*/*in vivo effect	Mechanism of action	Curcumin–radiation dosing/duration	References
More than additive effect of curcumin when combined with irradiation on viability of T98G and U87MG cells in vitro	Pro-apoptotic activity of curcumin via decreasing anti-apoptotic gene expression	25 μM curcumin 6 h prior to 5 Gy radiation	Dhapani et al. (2007)
Curcumin enhanced the radiation response on subcutaneous U87 glioma xenografts in vivo	Pro-apoptotic activity of curcumin via induction of DUSP-2 and therewith inhibition of ERK/JNK phosphorylation	50 mg/kg and irradiation (5 Gy) every 2 days, with curcumin 2 h. prior to radiation	Zhang et al. (2015)
Absence of a radiosensitizing effect of curcumin on clonogenic cell survival in U251 glioma cells in vitro	Curcumin inhibits cell proliferation. Clonogenic cell survival is reduced after 96 h. at doses exceeding 5 μM	5 μM curcumin 72 h prior to 1–6 Gy single dose and concomitant with 5 × 2 Gy fractionated radiation	Present study

Zhang et al. (2015) studied curcumin in combination with irradiation on subcutaneous U87 glioma xenotransplants, with a focus on dual-specificity phosphates (DUSPs). Oral administration of curcumin (50 mg/kg for 11 days) combined with irradiation (5 Gy) to tumour-bearing nude mice significantly delayed tumour growth by days 5–11 relative to non-treated controls. Curcumin alone did not affect tumour growth. No toxicity was reported (Zhang et al. 2015). The authors showed that DUSP-2 played an essential role in the regulation of apoptosis in U87 cells. DUSPs are cell-signaling enzymes that are known for their ability to control the activity and localization of MAPKs (Arnoldussen et al. 2009). MAPKs play a major role in regulating the signaling transduction of cell differentiation, cell proliferation, transformation, cell survival, and influencing cancer progression (Brennan et al. 2013; Narayan et al. 2018). Given that DUSP-2 shows a specificity for de-phosphorylation of MAPK, Zhang et al. (2015) demonstrated that DUSP-2 mRNA transcript levels were significantly upregulated following the combinatorial treatment, but not in the control groups. Also, the expression of DUSP-2 proteins was significantly increased in the combination group. Furthermore, they found a significantly reduced expression of the downstream effectors of MAPK, ERK, and JNK phosphorylation, in the combined groups, relative to the curcumin or irradiation alone control groups.

### Curcumin and radiation in GBM therapy

The dietary spice curcumin has been designated as promising therapeutic addition in brain tumour therapy, this on basis of reports on its anti-cancer efficacy, in particular for prostate cancer (e.g., Li et al. 2007; Liu et al. 2017). The present experimental data on clonogenic survival of U251 glioma cells confirm an anti-proliferative efficacy of curcumin in the lower dose range, and additional cytotoxicity after high curcumin dosages and long exposure duration. Our in vitro data on human glioma cells following single dose and fractionated irradiation with curcumin (cf. Figs. 2 and 3) additional to in vitro and in vivo preclinical data from the literature (Table 1) do not show a beneficial, radiosensitizing effect of curcumin in gliomas *at clinically achievable dose range.*

Nelson et al. (2017) reviewed a large number of preclinical and clinical publications on the efficacy of curcumin in medicine. Of all clinical trials that were done using curcumin in the treatment of several diseases, no double- blinded placebo-controlled trial has been successful. The authors provide evidence that curcumin is an unstable, reactive, non-bioavailable compound, and therefore a highly improbable lead (Nelson et al. 2017). Verma et al. (2016) reviewed the interaction of curcumin with radiotherapy on different cancer types. They notified ‘a general dearth of solid data’ and conclude that there is no clinical evidence of radiosensitization among the number of studies they reviewed. In view of the experimental data and emerging clinical observations presented here, there is also no evidence for *radiosensitizing efficacy of curcumin in GBM treatment*. Curcumin concentrations in the physiological range do neither exert an own cytotoxic effect nor show synergistic interaction with irradiation. Accordingly, clinical studies do not show any *anti-cancer* benefit from additional curcumin administration to glioma patients. Therefore, based on current scientific knowledge and literature, there is no justification to perform clinical trials in GBM patients aiming for radiosensitization using this combination treatment. However, it should be emphasized that curcumin has a wide variety of beneficial therapeutic activities, including anti-inflammatory, anti- and pro-oxidant, and chemo preventive activity which might be beneficial for cancer patients, as well (Hatcher et al. 2008; Hejazi et al. 2016).

Curcumin has an extremely low toxicity profile. Multiple clinical studies demonstrated that curcumin is well tolerated and safe (Clinicaltrials.gov 2019). Results from a pharmacokinetic Phase I trial by Cheng et al. (2001) suggested that curcumin is, however, not adequately absorbed from the gastrointestinal tract. The average peak serum concentration of curcumin was 1.77 μM after 8 g oral curcumin, which was found to be safe when taken daily for 3 months. Administration of a dose of 12 g was not feasible because of the bulky volume of the tablets. Hence, high orally taken doses of curcumin are not toxic and thus could be given safely to GBM patients, but, as explained above, no anti-cancer effects have to be expected. On the other hand, patients should be cautious taking large amounts of curcumin when they receive other therapeutic drugs as curcumin could influence the uptake of their regular medicine. Hussaarts et al. (2019) found that, in breast cancer patients, the exposure to tamoxifen and endoxifen was significantly reduced by concomitant use of curcumin, which could result in concentrations below threshold for efficacy. The authors recommend monitoring of tamoxifen plasma levels in patients using curcumin or to even stop curcumin intake during tamoxifen treatment (Hussaarts et al. 2019). It is unknown whether or not curcurmin influences the therapeutic efficacy of the alkylating agent temozolomide, the standard chemotherapy in GBM patients. Furthermore, the compound cannot cross the blood–brain barrier, which is a major barrier for most therapeutic agents to reach the tumour location (Van Tellingen et al. 2015; Sminia and Westerman 2016). Because of the heterogeneous BBB integrity in GBMs, varying from completely compromised in bulky tumour areas to slightly leaky in more invasive peripheral regions or even completely intact in sparsely invaded regions distant from the tumour bulk (Van Tellingen et al. 2015), it cannot be excluded that, in some tumour compartments, accumulation of curcumin might occur.

New approaches are being explored to improve the bioavailability of curcumin and/or to facilitate its transport over the BBB aiming to reach therapeutic levels at the tumour site. In this respect, recent advances in nano-based drug delivery systems offer a great opportunity (Patra et al. 2018). Kanai et al. (2012) demonstrated enhanced plasma curcumin levels in humans, hence improved bioavailability, after administration of curcumin encapsulated in nanoparticles. Micronized powder and, even more, liquid micelles of curcumin significantly improved the oral bioavailability of the compound in humans (Schiborr et al. 2014). The obvious crucial point is the intratumoural curcumin level that can be obtained. In an observational study on curcumin bioavailability (NCT01712542), concentrations and effects of orally administered micellar curcuminoids were determined in 10 GBM patients (Clinicaltrials.gov, 2019). The mean serum concentration of curcumin was 253 ng/ml (range 129–364), i.e. ~ 0.7 µM, and the mean intratumoural concentration was 56 pg/mg of tissue (range 9–151), which is approximately 0.15 µM [6]. Despite micellar administration, these concentrations were beyond therapeutic activity. Currently, there are no clinical trials with curcumin for GBM patients (Clinicaltrials.gov 2019).

Preclinical studies are ongoing to explore *functionalized*, i.e., chemically modified, nanoparticles for optimising their specificity, efficiency, and circulation time in blood, with the aim to increase intratumoural curcumin concentrations. Zhao et al. (2018) reported on targeted therapy of glioma cells in vitro and intracranial gliomas in mice using curcumin-loaded functionalized nanoliposomes. They demonstrated enhanced uptake and internalization of curcumin nanoliposomes that were modified by a brain targeting peptide RDP in U251 glioma cells, resulting in a cell cycle arrest at the S-phase and induction of apoptosis. The survival time of glioma bearing mice was significantly increased following repeated i.v. injection of curcumin nanoliposomes (Zhao et al. 2018). In a recent study, dual-targeted curcumin-loaded micelles were developed with the ability of crossing the BBB and targeting gliomas, in vitro and in vivo (Tian et al. 2019). They demonstrate these novel micelles functionalized with Tween 80 to move across the BBB, and both target and treat gliomas by combined effects of CD44-mediated endocytosis and glutathione-mediated intracellular release of curcumin (Tian et al. 2019). The use of functionalized nanoparticles loaded with curcumin is a very promising approach, but it is still questionable whether or not effective intratumoural concentrations would be achievable. Most perspective as adjuvant therapy to the current standard treatment of GBM patients might be expected from functionalized nanoparticles loaded with a combination of curcumin and/or other targeted—by preference—radiosensitizing anti-cancer drugs (Bikhezar et al. 2020).
